# Prenatal Vitamin D Deficiency and Maternal and Fetal Health Outcomes

**DOI:** 10.7759/cureus.69508

**Published:** 2024-09-16

**Authors:** Lilia Tsenkova-Toncheva, Eleonora Hristova-Atanasova, Georgi Iskrov, Rumen Stefanov

**Affiliations:** 1 Social Medicine and Public Health, Medical University of Plovdiv, Plovdiv, BGR; 2 Institute for Rare Diseases, Plovdiv, BGR

**Keywords:** asthma, autism, gestational diabetes, low birth weight, preconception vitamin d deficiency, preeclampsia, preterm birth, vitamin d deficiency, vitamin d recommendations, vitamin d supplementation

## Abstract

Vitamin D deficiency (VDD) is a significant health issue that could have serious implications for the well-being of women and their offspring. Prenatal vitamins are widely used, but deficiency still occurs frequently in the preconception period, during pregnancy, and in breastfed infants.

We analyzed the association between prenatal VDD and maternal and fetal health outcomes by reviewing studies conducted in Europe. The literature was searched for articles published in the last 10 years focusing on preeclampsia, gestational diabetes mellitus, preterm birth, low birth weight, asthma, and autism spectrum disorder. We identified a total of 43 review articles, 31 original articles, and two guidelines.

During pregnancy, VDD is associated with a higher likelihood of developing gestational diabetes mellitus and preeclampsia. It may also lead to an increase in the risk of preterm birth, low birth weight, as well as asthma, and autism spectrum disorder in the offspring. While the official guidelines for vitamin D dosage differ in various countries, health authorities usually recommend a total daily supplement intake of 400-2,000 IU.

In conclusion, this review emphasizes the importance of establishing guidelines for vitamin D supplementation as well as the requirement of official standards for the consumption of vitamin D in the prenatal period. Future research should concentrate on developing more unified approaches to vitamin D assessment and establishing preventative measures that can be incorporated into prenatal care programs.

## Introduction and background

Vitamin D deficiency (VDD) is recognized as a worldwide public health concern, despite being preventable [1]. Nutritional guidelines recommend adequate dietary intake of vitamin D; however, many people across various countries still fail to meet the recommended levels [2,3]. This deficiency is particularly prevalent during pregnancy and represents a significant health issue that could have serious implications for the well-being of both women and their offspring; therefore, it is a topic of increasing attention [3-5]. Research indicates that the prevalence of VDD in pregnant women ranges from 26% to 98% in many countries worldwide. Despite the widespread use of prenatal vitamins, deficiency still occurs frequently during the preconception period, pregnancy, and in breastfed infants [6].

Vitamin D is a fat-soluble vitamin that is vital for musculoskeletal health and has been recognized to be useful for the prevention and treatment of rickets and osteomalacia [7-9]. In addition to its essential role in maintaining bone health throughout life, vitamin D plays several important roles in immune system development, brain development, cell differentiation, and fetal lung development [10,11]. Several studies highlight the potential role of vitamin D for female fertility, as it stimulates the synthesis of estrogens and progesterone. Furthermore, it is found to play a role in the development of the uterus and endometrium, which is crucial for implantation in the preconception period [12,13].

During pregnancy, the fetus depends entirely on the mother’s vitamin D reservoir, which explains the correlation between mother and cord blood vitamin D concentration [3,14]. Several unfavorable maternal outcomes, including preeclampsia, primary cesarean section, and gestational diabetes mellitus (GDM), are linked to low maternal vitamin D levels throughout pregnancy [6,15-17]. Furthermore, fetal intrauterine development restriction and a number of adverse fetal and neonatal health outcomes, such as an increased risk of preterm birth (PTB), abortion, low birth weight, and neonatal hypocalcemia, have been connected to prenatal VDD [18,19]. Long-term pathologies potentially arising from maternal-fetal VDD include mineralization defects, bronchial asthma, autoimmune and atopic diseases, and neurodevelopmental disorders [20,21].

As VDD has been linked to a number of negative health consequences, the best possible pregnancy outcomes and fetal and maternal health all depend on the antenatal vitamin D state [22]. Future studies are needed in order to find practical solutions for raising pregnant women's vitamin D levels. For many pregnant women, vitamin D supplements may be recommended throughout pregnancy, which should serve as the foundation for adjusting public health initiatives aimed at lowering the illness burden and enhancing vitamin D intake for both mothers and infants [23].

The aim of this review is to determine the relationship between prenatal vitamin D and various health outcomes for both the mother and the newborn, as well as to review national recommendations for vitamin D dietary references and supplementation for pregnant and lactating women in European Union countries.

## Review

This literature review is developed following the six standard methodologies described by Templier and Paré [24]. A comprehensive study of research papers published in the last ten years was conducted to examine the relationship between maternal vitamin D insufficiency during pregnancy and adverse health outcomes. Our selection included empirical research or conceptual papers that initiated a specific path of investigation, modified the formulation of problems or concerns, introduced innovative methodologies or concepts, or generated substantial discussion. Our study involved an extensive electronic search across several databases, such as Web of Science, MEDLINE/PubMed, Google Scholar, and Cochrane, covering the period from January to June 2024. The search utilized the designated terms: vitamin D deficiency, preconception vitamin D deficiency, vitamin D supplementation, gestational diabetes, preeclampsia, preterm birth, low birth weight, asthma, autism, vitamin D recommendations, and pregnancy. Furthermore, we assessed the publications to determine their relevance and to foster impartiality while avoiding prejudices or mistakes. The inclusion criteria specifically targeted research papers, reports, and studies that examined the effects of maternal vitamin D deficiency (VDD) during pregnancy on health outcomes and associated recommendations. These studies included case-control, cross-sectional, cohort, and prospective observational designs. The evaluation approach encompassed the examination of both qualitative and quantitative research, together with the analysis of gray literature. The selected sources encompass official written materials, current recommendations from executive entities, and peer-reviewed journals. The collections consisted of materials in both the English and Bulgarian languages. Included in the exclusion criteria were animal experiments, case reports, and conference articles. Excluded from consideration were papers published before 2014, materials written in languages other than English, and works that did not address vitamin D insufficiency in relation to pregnancy.

Preconception VDD

Research indicates that VDD is prevalent among preconception women, particularly workers and nulliparous women [25,26]. Nevertheless, the majority of study literature has concentrated on vitamin D in individuals who are undergoing assisted reproductive procedures, with few studies examining the influence of preconception vitamin D concentrations on pregnancy outcomes [27]. Vitamin D status is commonly assessed by measuring serum 25-hydroxy vitamin D (25(OH)D) concentration, with several European organizations, including the European Food Safety Authority, adopting a threshold of 50 nmol/L as a sufficient level [28]. Multiple studies have demonstrated that women with higher 25(OH)D concentrations had a higher likelihood of becoming pregnant [29-31]. In central North Carolina, a study involving 522 fertile women found that those with serum vitamin D levels of at least 50 ng/mL had an estimated 35% increase in fecundability, while those with levels below 20 ng/mL had an estimated 45% decrease in fecundability, which is the likelihood of conception within a month or menstrual cycle [30]. According to a distinct investigation that involved 1191 American women who had previously experienced miscarriages, a higher rate of conception was associated with higher 25(OH)D concentrations [31]. Recent research suggests that the mother's preconception serum 25(OH)D level can be used to predict the pregnancy outcome of in vitro fertilization (IVF) therapy. Additionally, a preconception serum 25(OH)D level of at least 50 nmol/L is associated with an increased likelihood of a successful IVF treatment-related pregnancy [32]. Zhang et al. conducted one of the rare studies to investigate vitamin D and fecundity in both preconception women and males. The growing corpus of research that emphasizes the importance of preconception vitamin D for fertile couples pursuing natural conception is further illustrated by the positive correlation between the increased rate of conception and adequate vitamin D among preconception men. This data also corroborates the necessity of revising and improving pre-pregnancy healthcare guidelines [25]. Additionally, the risk of miscarriage is reduced and the likelihood of delivering a live male infant is increased when preconception vitamin D levels are elevated [33]. Research has established the significance of obtaining an adequate amount of vitamin D daily prior to conception. However, it has also been shown that taking an excessive amount of the vitamin before becoming pregnant may have a detrimental effect on fertility, the quality of embryos, and perinatal outcomes, particularly in women who plan to conceive through assisted reproductive technology, as preconception care is more obtainable to them. Both the cumulative detrimental effects and the advantages of vitamin D should be considered, as it is a fat-soluble vitamin [34,35].

Gestational diabetes mellitus

The chronic pregnancy condition known as gestational diabetes mellitus (GDM), defined as hyperglycemia first detected during pregnancy, is prevalent among millions of women globally and is associated with an increased likelihood of complications [36,37]. Research has established a significant connection between VDD and an increased risk of developing gestational diabetes mellitus [17]. A clinical experiment conducted on 120 women who were less than 12 weeks pregnant revealed that the administration of 50,000 IU of vitamin D every two weeks had a substantial positive effect on the improvement of insulin resistance [38]. An investigation conducted by Shang et al. found that insufficient levels of 25(OH)D3 serve as a dependable marker for gestational diabetes mellitus. The study revealed a negative association between fasting blood glucose levels during the first trimester and 25(OH)D3 levels [39]. The systematic analysis conducted by Zhang et al. includes 25 randomized controlled trials and 87 observational studies with a total sample size of 55,859 and 2,445 women. The findings of this research suggest that elevated blood vitamin D levels may increase the likelihood of developing gestational diabetes mellitus. The analysis further indicated that the administration of vitamin D supplements during pregnancy could enhance the manifestation of gestational diabetes mellitus [40]. Another analysis found that providing maternal vitamin D supplementation during pregnancy to women with varying nutritional status decreases Homeostatic Model Assessment for Insulin Resistance (HOMA-IR) levels but does not affect fasting plasma glucose levels. The recommended dosages range from 200 IU per day to 300,000 IU once [41]. In addition, Sadeghian et al. found that with every 4 ng/mL increase in circulating 25(OH)D, the likelihood of getting GDM decreases by 2%. Moreover, the risk of acquiring GDM in the highest category of 25(OH)D levels is 29% lower compared to the lowest group [42].

Preeclampsia

Preeclampsia, eclampsia, gestational hypertension, chronic hypertension with preeclampsia, and chronic hypertension are among the conditions collectively referred to as pregnancy-induced hypertension (PIH), which is characterized by poor blood pressure control throughout pregnancy [6]. Preeclampsia is a highly concerning pregnancy complication, typically manifesting as new-onset hypertension and proteinuria in the third trimester, and can quickly escalate to severe outcomes, including the death of both mother and fetus [43]. Numerous studies have found an association between VDD and preeclampsia [44-46]. Research suggests that patients with pregnancy-induced hypertension often exhibit low vitamin D levels, indicating a higher prevalence of VDD among these individuals and suggesting it as a possible risk factor for the onset of pregnancy-induced hypertension [6]. Moreover, a meta-analysis conducted by Khaing et al., which included 27 randomized controlled studies involving 28,000 women, revealed that the administration of calcium, vitamin D, or a combination of both significantly decreased the likelihood of preeclampsia when compared to a placebo [47]. An analysis conducted by AlSubai et al. examined 34 studies, consisting of 10 randomized controlled trials and 24 observational studies. The findings indicate that low maternal 25-hydroxyvitamin D levels are associated with a higher risk of preeclampsia. However, supplementing with 25-hydroxyvitamin D reduces the occurrence of preeclampsia [48]. These results emphasize the possible advantages of vitamin D supplementation in avoiding preeclampsia, indicating that it should be considered as an intervention approach to manage pregnancy-induced hypertension [49,50].

Negative outcomes in newborns and prenatal VDD

Preterm Birth and Low Birth Weight

Premature birth, defined as delivery before 37 weeks gestation, is the leading cause of death among infants under one-year-old and has been linked to VDD during pregnancy in numerous studies [51]. The vitamin D receptor (VDR) gene encodes the receptor for vitamin D, which mediates its effects. Variants in VDR lead to differences in vitamin D levels and a higher chance of premature birth [52]. A pregnancy-related 25(OH)D deficit would raise the risk of small for gestational age (SGA), preterm birth (PTB), and low birth weight (LBW) [44]. A population-based prospective cohort study conducted in Rotterdam, Netherlands, by Miliku et al. found that mothers with lower 25(OH)D levels had children with increased fetal growth restriction during the third trimester. This restriction led to smaller head circumference, shorter body length, and lower birth weight (all P < 0.05) compared to mothers in the highest quartile. Moreover, those with 25(OH)D levels in the lower quartile had a higher probability of experiencing preterm birth (OR: 1.72; 95% CI: 1.14, 2.60) and having children that were small for their gestational age [53]. Additionally, maternal VDD greatly raises the likelihood of preterm birth (P = 0.001), whereas a systematic study by Palacios et al. found that pregnant women can get adequate serum vitamin D levels by taking 2000 IU of vitamin D supplements [50]. A comprehensive analysis conducted by Lian et al., comprising of seven cohort studies, 13 case-control studies, and four cross-sectional studies, revealed that it is important to monitor vitamin D levels during the second trimester of pregnancy. If necessary, vitamin D supplements should be provided, as VDD during pregnancy is likely to significantly impact preterm birth mortality [54].

Asthma

Asthma, a prevalent chronic non-communicable disease affecting both children and adults, is characterized by fluctuating respiratory symptoms and intermittent airflow obstruction [55]. According to numerous studies, maternal vitamin D concentration during pregnancy may affect lung development and the likelihood of childhood wheezing and asthma [44,56]. In the Vitamin D Antenatal Asthma Reduction Trial, pregnant women who were at risk of giving birth to children with asthma were randomized to receive either 4400 international units/d of vitamin D or a placebo + 400 international units/d of vitamin D. Up until age three, asthma and persistent wheezing were recorded. Maternal vitamin D supplementation, especially in mothers with baseline 25(OH)D levels above 30 ng/mL, decreased the incidence of asthma and recurrent wheezing in their children up to the age of three. This suggests that early pregnancy and higher vitamin D status are important for the prevention of asthma and recurrent wheezing in children [57,58]. Grant et al. did a study that found that supplementing with vitamin D3 reduces sensitivity to aeroallergens during pregnancy and very early infancy. The researchers administered either a placebo or one or two daily oral vitamin D doses to pregnant women from 27 weeks of gestation until delivery and thereafter to their infants from birth until six months of age. At 18 months of age, the children underwent an assessment to determine the presence of serum-specific IgE antibodies. Evidence has shown that the administration of vitamin D supplements during pregnancy and in newborns reduces the percentage of children who become sensitive to mites by the age of 18 months. Furthermore, there were variations among the research groups in the proportion of children whose primary care visits involved a medical diagnosis of asthma from their treating physicians [59].

Autism Spectrum Disorder

Autism spectrum disorder (ASD) is a neurodevelopmental disorder marked by impairments in social communication and the presence of restricted, repetitive behaviors or interests [60]. Research has indicated that ASD is associated with vitamin D status. Multiple studies have shown that children and adolescents diagnosed with autism exhibit decreased levels of vitamin D [61-63]. This phenomenon is also exemplified in siblings, where the child with autism exhibits a reduced level of 25(OH)D compared to their siblings [64]. Moreover, a meta-analysis of prospective studies conducted by Wang et al. found that a 54% higher risk of ASD in children was associated with a deficiency in maternal or neonatal vitamin D (OR: 1.54, 95% CI: 1.12; 2.10, p = 0.0071, I2 = 81.2%) [65]. The Netherlands-based prospective cohort study done by Vinkhuyzen et al. aimed to examine the association between prenatal vitamin D levels and the likelihood of developing ASD in children. An analysis of 4334 children and their mothers, out of whom 68 were diagnosed with ASD, revealed that prenatal 25(OH)D insufficiency during mid-gestation is associated with a risk of being diagnosed with ASD that is more than double twice as high. Thus, it may be feasible to decrease the occurrence of autism in children by administering prenatal nutrition in a manner that is both safe and cost-effective [66]. Furthermore, Stubbs et al. presented encouraging findings, indicating that the administration of vitamin D supplements to pregnant women is linked to a reduced probability of their offspring having autism. A dosage of 5000 IU/d of vitamin D3 was administered to pregnant women who had previously had a child with autism. Infants were then given a daily supplementation of vitamin D3 at a dosage of 1000 IU for their first three years of life. This group of children was monitored at the ages of 18 and 36 months. The findings indicated that one in every 19 children, or 5%, had autism, whereas the total likelihood of recurrence was 20% [67].

National recommendations for vitamin D dietary references for pregnant and lactating women in European Union countries

We have compiled a timeline encompassing several European Union countries, which provides the latest guidelines for vitamin D dietary reference values during pregnancy. Our primary focus is on data from Europe, as European populations have similar health behaviors that are influenced by geographical, historical, and cultural factors. The health agencies of each country give these guidelines, which include doses indicated in both International Units (IU) and micrograms (µg) (Table 1).

**Table 1:** National recommendations for vitamin D dietary references for pregnant and lactating women in European Union countries a: Deutsche Gesellschaft für Ernährung – Österreichische Gesellschaft für Ernährung – Schweizerische Gesellschaft für Ernährungsforschung – Schweizerische Vereinigung für Ernährung (German Nutrition Society, Austrian Nutrition Society, Swiss Society for Nutrition Research, Swiss Nutrition Association) b: Agence nationale de sécurité sanitaire de l'alimentation, de l'environnement et du travail (French Agency for Food, Environmental and Occupational Health and Safety Organisation) c: Food Safety Authority of Ireland IU: International Units; µg: micrograms

Country	Authority	Recommended dose in IU	Recommended dose in µg
Austria	DACH ^a ^[68]	800	20
Belgium	Hoge Gezondheidsraad [68]	800	20
Bulgaria	Ministry of Health of Bulgaria [69]	600	15
Denmark	Nordic Council of Ministers [68]	400	10
Finland	Nordic Council of Ministers [68]	400	10
France	ANSES ^b ^[70]	400	10
Germany	DACH ^a ^[71]	800	20
Iceland	Nordic Council of Ministers [68]	400	10
Ireland	FSAI ^c ^[68]	400	10
Italy	Ministry of Health of Italy [72]	400	10
Netherlands	Health Council of the Netherlands [68]	400	10
Norway	Nordic Council of Ministers [68]	400	10
Poland	Polish National Food and Nutrition Institute [73]	600	15
Spain	Instituto de Nutrición y Tecnología de los Alimentos [73]	600	15
Sweden	Nordic Council of Ministers [68]	400	10
Switzerland	DACH ^a ^[68]	800	20

It is demonstrated that the recommended doses of vitamin D for pregnant women in Europe range from 400 IU (10 µg) to 800 IU (20 µg), with variations reflecting regional differences in sunlight exposure, dietary habits, and health policies. Countries like Germany, Austria, and Belgium recommend higher doses (800 IU) to account for lower sunlight exposure and dietary intake, while others like France, Italy, and the Nordic countries suggest 400 IU. Spain, Poland, and Bulgaria opt for an intermediate dose of 600 IU. These variations underscore the need for tailored public health measures to ensure adequate maternal vitamin D levels across diverse European Union contexts [68-75].

In order to achieve recommended dietary doses of vitamin D, pregnancy multivitamin pills are widely used in most countries but often contain just 200-400 IU of vitamin D. For the majority of people who get enough sun exposure, this dosage is appropriate; however, it is inadequate to address VDD. Several health organizations recommend taking supplements containing at least 400 IU daily and consuming 1,000-2,000 IU daily overall from both food sources and supplements [76].

According to the recommendations of the Ministry of Health in Bulgaria, pregnant women who do not spend enough time outdoors are at risk of VDD; therefore, some women may benefit from supplementing with calcium and vitamin D. The optimal prescribed daily intake of vitamin D for pregnant and breastfeeding women is 15 micrograms or 600 IU. The Bulgarian Society of Endocrinology, however, advises that supplementation with vitamin D in pregnant women should be done under the control of 25(OH)D to maintain optimal levels between 30-50 ng/ml. In cases where measuring the levels of 25(OH)D is not feasible, the suggested daily dosage for pregnant and lactating women is 2000 IU daily [69,75].

## Conclusions

Hypovitaminosis D has emerged as a widespread public health issue, particularly for pregnant women due to their increased vulnerability. Numerous studies have shown that it may be crucial for preserving mother-fetal health throughout the prenatal and postnatal phases and preventing negative consequences. Due to its immunomodulatory, skeletal, and calcium regulatory properties, as well as its fertility effects, vitamin D not only improves the prognosis of preeclampsia, gestational diabetes, and conception rates in mothers, but it also has a direct impact on respiratory disorders, fetal weight at birth, and neonatal neurological development.

Most European Union countries advocate for vitamin D supplementation due to its potential benefits for pregnant and lactating women; however, the recommended dosages vary, which may be due to differences in dietary habits, sunlight exposure, and healthcare strategies.

This review underlines the need for developing more consistent guidelines for vitamin D supplementation and the necessity of official standards for vitamin D intake during the preconception period and throughout pregnancy. Future studies should focus on creating more standardized methods for assessing vitamin D levels and on creating preventative strategies in order to improve public health initiatives that support healthy pregnancies and pregnancy outcomes.
